# Phylogeny of Diving Beetles Reveals a Coevolutionary Arms Race between the Sexes

**DOI:** 10.1371/journal.pone.0000522

**Published:** 2007-06-13

**Authors:** Johannes Bergsten, Kelly B. Miller

**Affiliations:** 1 Entomology Department, Natural History Museum, London, United Kingdom; 2 Division of Biology, Imperial College London, Silwood Park Campus, Ascot, United Kingdom; 3 Department of Entomology, Museum of Southwestern Biology, University of New Mexico, Albuquerque, New Mexico, United States of America; University of Exeter, Cornwall Campus, United Kingdom

## Abstract

**Background:**

Darwin illustrated his sexual selection theory with male and female morphology of diving beetles, but maintained a cooperative view of their interaction. Present theory suggests that instead sexual conflict should be a widespread evolutionary force driving both intersexual coevolutionary arms races and speciation.

**Methodology/Principal Findings:**

We combined Bayesian phylogenetics, complete taxon sampling and a multi-gene approach to test the arms race scenario on a robust diving beetle phylogeny. As predicted, suction cups in males and modified dorsal surfaces in females showed a pronounced coevolutionary pattern. The female dorsal modifications impair the attachment ability of male suction cups, but each antagonistic novelty in females corresponds to counter-differentiation of suction cups in males.

**Conclusions:**

A recently diverged sibling species pair in Japan is possibly one consequence of this arms race and we suggest that future studies on hypoxia might reveal the key to the extraordinary selection for female counter-adaptations in diving beetles.

## Introduction

Sexual conflict in mating systems, due to differences in investment and direct mating costs, can lead to intersexual arms races [1], [2] as well as being a potential engine of speciation [3], [4]. Rapid evolution of male adaptations and female counter-adaptations [1], [5] explicitly predicts that changes in male and female characters should be correlated as coincidental transformations on internal branches of a phylogeny. However, despite claims of the widespread occurence of sexual conflict fuelling arms races [1], credible empirical examples from a phylogenetic perspective, are largely lacking and the major question today is whether sexual conflict really generates sexually antagonistic evolution [6], [7]. *Drosophila* provide a model system on a microevolutionary scale [2], and in particular, artificial selection experiments have convincingly demonstrated sexual antagonistic coevolution [8], [9] involving accessory gland substances [some 80 different Acps] in the male ejaculate that in various ways affect the female [10]. The exponentially increasing literature on sexual conflict involving various organisms includes population crosses, mating experiments, meta-analyses of groups with different mating systems, comparisons of evolutionary rates in sexual versus asexual characters, theoretical models, experimental manipulations of sexual traits and studies of the economics and costs of matings and all contribute to the growing body of evidence [1], [2]. Indeed a full-scale Kuhnian paradigm shift is starting to be acknowledged [11] where traditional sexual selection in terms of male-male competition and female choice, rather than being the golden standard for interpreting mating interactions and outcomes, merely becomes “*an interesting emergent property of a more ancient and far-reaching evolutionary dynamic: sexual conflict*” [12]. But the paradigm shift is not friction free and controversies remain, in particular centred around what the alternatives predict and thus what data is really needed to corroborate sexual conflict as oppose to traditional sexual selection [13]–[15] something perhaps not as straight forward as previously believed. However the claim that the substantial direct costs of multiple matings to females can be compensated for by their offspring acquiring indirect genetic benefits or “good genes” has been questioned by recent studies [16], [17].

On a macroevolutionary level examples are scarce, but water striders (Gerridae) [18] and hermaphroditic snails [19] provide the only putative example of correlated characters involved in a sexual arms race. Since macroevolutionary tests of coevolutionary scenarios require a phylogenetic context, incomplete taxon sampling and uncertainty in phylogenetic reconstruction often undermine the credibility of empirical examples. Recently developed Bayesian applications to phylogenetics offer new possibilities to test evolutionary scenarios while accounting for the uncertainty in phylogenetic reconstruction [20]–[22]. In addition, thorough taxonomic sampling in phylogenetic studies have proved to be of fundamental importance for accurate reconstruction [23] as well as answering various questions on evolution and speciation [24]. Here we provide an example of an antagonistic intersexual arms race, where confounding effects of incomplete taxon sampling is minimized and phylogenetic uncertainty statistically accounted for.

**Figure 1:**
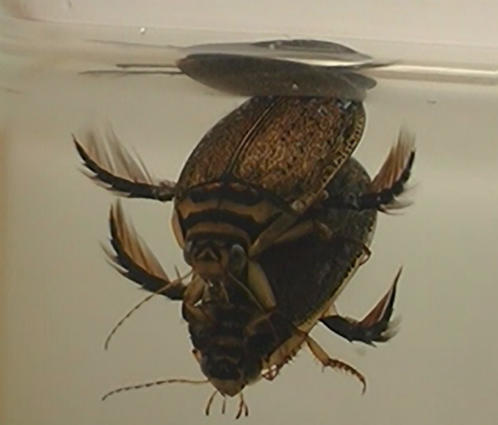
A mating pair of *Acilius sulcatus.* The male on top has access to air with the abdominal apex and can replenish the air supply kept in the subelytral space [30]. The female below the surface is at this stage beyond reach of atmospheric oxygen.

Diving beetles (family Dytiscidae) are predatory aquatic insects. Sexual conflict in diving beetles has been suggested to explain various dorsal modifications in females [25], [26]. The mating system is typical for sexual conflict scenarios that involve substantial direct costs [1]; with no courtship, males simply attack bypassing females, females resist behaviourally by attempting to dislodge the male in rapid and erratic swimming moves [27, Video S1], and mating interactions are characterized by a very long postcopulatory guarding phase [27]. To grab the female, males have their front feet modified into a palette equipped with suction cups that are attached to the female dorsal surface at initial contact [28]. A possible unique mating cost to females relates to the fact that these secondarily aquatic insects are dependent on atmospheric oxygen, which is carried under the elytra and replenished with frequent trips to the surface (normally once every 8–15 minutes [29]). Only the male however, has access to air during the mating (Fig. 1). Indeed, precopulatory violent shaking by the male seems to increase the female's need for oxygen which is followed by copulation when the female is exhausted and non-resisting [30]. During the approximately six hours of postcopulatory guarding in the diving beetle *Dytiscus alaskanus*, the male periodically tilts the female upward allowing her to replenish the air supply [27]. The evolution of this behaviour further emphasizes the importance of this cost to females, although it has yet to be experimentally quantified. An alternative explanation for the resistance behaviour of females is the classical female choice [13]–[15], where, although the behaviour may be costly, this is compensated for through good genes passed on to her offspring since only fit males able to withstand her resistance will fertilise her eggs. Until unambiguously tested with breeding experiments and fitness measures in offspring such a scenario cannot be excluded, but more and more evidence suggest that this is unlikely to compensate for the substantial direct costs [2], [16], [17].

To trace the evolutionary history of sex-specific characters in diving beetles, we used the recently revised genus *Acilius* which contains 13 extant species distributed over the Northern Hemisphere [31]. The genus is characterized by having dense macropunctures on the dorsal surfaces, females with prominent, setose furrows on the elytra, and males with broadly expanded protarsi equipped ventrally with three large and many minute suction cups. These structures even attracted the attention of Charles Darwin, who regarded the setose female furrows in *Acilius* as an example of an aid for males to better grip females during mating [32]. However, it is clear from basic physical laws and simple experiments [Supporting Text S1], that the mechanically working male suction cups function best on smooth surfaces where complete contact around their circumference enables attachment.

## Results and Discussion

**Figure 2:**
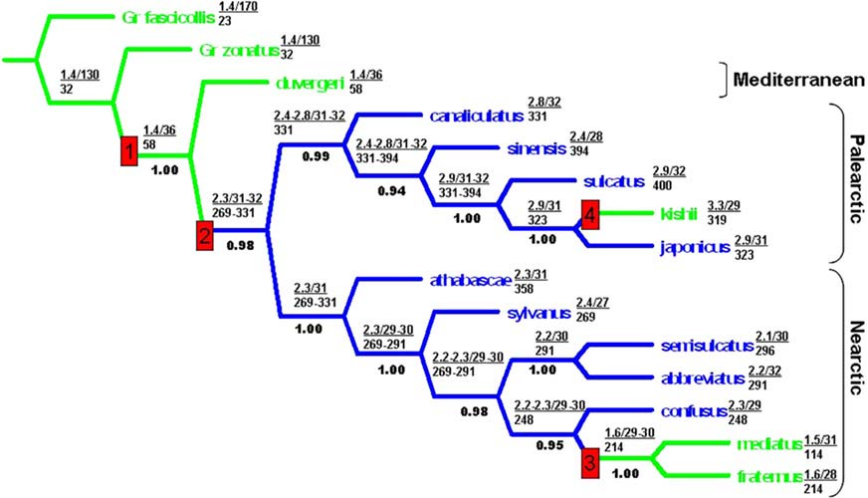
Phylogeny of *Acilius* based on 1693 characters. Posterior probability of the topology = 0.85, from Bayesian analysis. Treelength = 793 steps from parsimony search (single best cladogram). Values below branches are clade support values (posterior probabilities). Values above branches are optimized continuous valued characters of male suction cups (S1–S4: see fig. 4D); (S1 as a fraction of S3/S4 in µm above, number of S4 cups below). Colours on branches are optimized female condition; green = non-setose, blue = with setose furrows. Visualization of the major character transformation events 1–4 [in red] see fig 3.

We analyzed one mitochondrial gene (Cytochrome oxidase I), two nuclear genes (Histone III and Wingless) and morphological characters to infer the evolutionary relationship of the 13 *Acilius* species. Bayesian and parsimony analysis on the concatenated data converged on the same, fully resolved phylogeny shown in Fig. 2, with very high clade support values (posterior probabilities: 0.94–1.0). Although missing DNA data from the possibly extinct Chinese species *A. sinensis*, the morphological character matrix (Tab. S1, S2) strongly inferred the placement of this species in the phylogeny. Palearctic and Nearctic species groups are clearly supported as monophyletic with the exception of the Mediterranean species *A. duvergeri*, which is sister to all other *Acilius*. Bayesian posterior probability of this topology is 0.85 with the next best topology 0.05. Moreover, only three additional topologies from the Bayesian analysis together make up a 95% credible set of trees (cumulative posterior probability>0.95). This is interpretable as the true tree being among these four topologies with a probability of 0.95, assuming the model is correct [33]. Accomodating the phylogenetic uncertainty, ancestral character reconstructions were carried out on all four topologies and inferred coincidental character transformations discussed below are unaffected by their topological variation.

**Figure 3:**
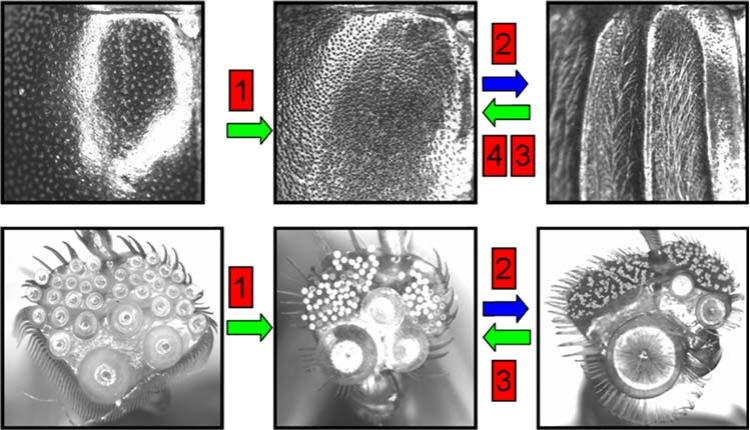
Major coevolutionary events in the intersexual arms race of *Acilius.* Top row: close-up of female cover wings, bottom row: male front foot with suction cups. 1: The ancestor of *Acilius* evolved a back with dense and large punctures accompanied in males by large cup size differentiation into three large and multiple smaller cups. 2: Females evolve setose furrows, males further differentiate their suction cups, one very large, two medium sized and radically increasing the number of minute cups. 3: The last transformation reverse in both sexes supporting the theory that arms races can both escalate and de-escalate [43]. 4: The female, but not male, condition also reverses in the recent species *A. kishii*.

Dorsal macropunctures and female setose furrows show transformations at the ancestor of *Acilius*, at the ancestor of all taxa except *A. duvergeri*, at the ancestor of the sister taxa *A. mediatus* and *A. fraternus* and at the terminal branch, *A. kishii* (Figs. 2–3). Reconstruction of size and numbers of male suction cups show major coincidental character transformations on the same ancestral nodes except the branch subtending *A. kishii*. Thus the male of the ancestral lineage that developed the dorsal macropunctures, radically evolved from having a set of few medium sized suction cups to having three large and multiple minute suction cups (1:Fig. 3). On the next internode the evolution of female setose elytral furrows coincides with a second major male transformation where the suction cups further differentiated into one basal very large suction cup, two medium sized and five times the number of minute cups (2:Fig.3). Finally, the loss in females of setose furrows in the ancestor to *A. fraternus*/*A. mediatus* coincides with a reversal in male characteristics resembling the initial condition before females changed (3:Fig.3); differentiation among the three larger cups is nearly completely reversed, whereas the number of minute cups decreases only moderately in the ancestor, but further in *A. mediatus*. The pattern mimics results from a comparative population-level study [25] where male suction cups increase in differentiation as a response to increasing frequency of a granular female morph of the diving beetle *Graphoderus zonatus*.

**Figure 4:**
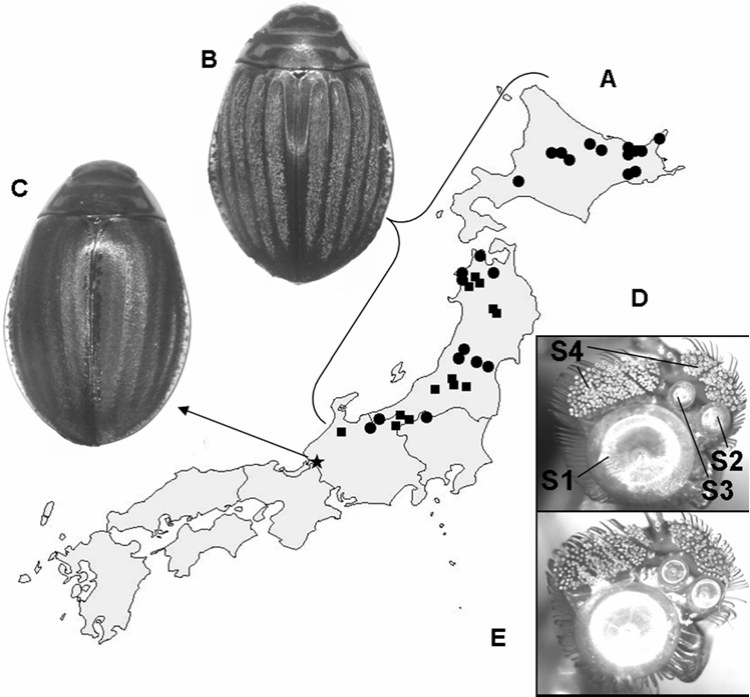
A possible example of recent allopatric speciation driven by sexual conflict [3]. A: The distribution of *A. japonicus* and *A. kishii* in Japan [31]. • = specimens examined and ▪ = literature records of *A. japonicus,* ★ = *A. kishii*. B: ♀ of *A. japonicus*. C: ♀ of *A. kishii*. D: ♂ front foot of *A. kishii* indicating the labelling of suction cups S1–S4. E: ♂ frontfoot of *A. japonicus*. The sibling species pair *A.kishii* and *A. japonicus* are endemic to Japan, with *A. kishii* occurring in a single mountain lake in Fukui prefecture, just south of *A. japonicus* range. They differ morphologically only in secondary sexual characters involved in the arms race, in particular the loss in *A. kishii* of setose furrows in the female.

However, coincidental character transformations at the same ancestral nodes cannot indicate which character evolved first and which was the response. An independent line of evidence regarding the order of coevolution exists in the recently diverged species *A. kishii.* This species is confined to a single mountain lake and population in Fukui prefecture, Japan [31]. Females of this species have secondarily lost the setose elytral furrows, although slight impressions of furrows are still apparent (Fig. 4). Males of *A. kishii* are very similar to the sibling species *A. japonicus* (Fig. 4) but ratio comparisons between the three largest cups show that S1 is relatively larger compared to S2 and S3 in *A. kishii* (MANOVA, P<0.01, n = 7, Tukey's HSD test). However, the basic pattern of one very large cup, two medium sized and a large number of minute cups, characterising all other species with setose females, also characterises males of *A. kishii* (Fig. 4). This provides the only direct evidence of the order of evolution in the *Acilius*-arms race; suction cup differentiation in males is the response to previous changes in female dorsal surfaces. The advantage of differentiating the cups into smaller and larger ones as the surface evolves sculpture can tentatively be explained by the equations defining the suction force and attachment time [Supporting Text S1]. Both are influenced most by the radius of the cup and the radius of the leaking channels; small cups can increase the suction force and attachment time by reducing the leaking channels, while larger suction cups achieve the same effect by increasing the volume and area of the cups.

In addition, the *A. japonicus*/*kishii* species pair may be an example of speciation at an early stage driven by sexual conflict. At least this singular example fulfils the prediction that characters involved in the arms race change faster than others [5] (e.g. male genitalia are identical while being diagnostically different between all other *Acilius* species [31]). DNA similarity of CO1 between *kishii* and *japonicus* (99.5–99.6%), lies in the range of the within species variation for *A. japonicus* (99.5–99.8% for three sequenced individuals). Branch length estimates from the Bayesian analysis also illustrate the recent history of the split (Fig. S1). At present the ranges of these two taxa are completely allopatric, but this disjunction certainly dates from the withdrawal northwards of the last glacial advance during which northern biota expanded further south in Japan [34]. Thus, it is expected that the isolation dates from the Holocene and speciation is likely to have occurred in a few thousand generations, whereas theory on sexual conflict suggests speciation can occur in even less time [3].

Quantifying the cost to females of multiple matings is a challenge for future studies and we tentatively predict that in the natural history of diving beetles, aquatic insects dependent on atmospheric oxygen, lies the key to the extraordinary female counter-adaptations in *Acilius* and other diving beetles. While the actual cost for females of multiple matings is unknown, observations on *Acilius* from the field reveal a very high male harassment rate [35]. Likewise, while the biomechanical effect of a structured surface for suction cups can be predicted from physical laws [Supporting Text S1] or simple measurements on dead animals [28], only experimental mating trials will reveal the net effect when compensatory behaviour can interact with the equation.

## Materials and Methods

### Taxon selection and molecular methods

Outgroup exemplars include representatives from the genus *Graphoderus*, the sister group to *Acilius* [26]. *Acilius* exemplars include all 13 extant species for morphology and all except *A. sinensis* for sequence data. Genomic DNA was extracted from legs and thoracic muscle tissue from ethanol-preserved material collected between 2000 and 2003 using standard protocols. DNAs and controls were amplified using PCR and a DNA Engine DYAD Peltier Thermal Cycler using the primers *Haf* and *Har* (for *histone III*) [36], *LepWg1* and *LepWg2a* (for *wingless*) [37], and *C1-J-2183* and *TL2-N-3014* (for *cytochrome oxidase I*) [38]. Product yield, specificity of amplification and contamination were investigated using agarose gel electorphoresis. PCR products were purified and cycle sequenced using ABI Prism Big Dye (version 3). Sequencing reactions were column purified and fractionated with an ABI 3730xl DNA analyser (DNA Sequencing Center, BYU). Fragments were sequenced from complementary strands and these were examined and edited using the program Sequencher. Genebank accession numbers are given in Table S3. A morphological matrix of 46 discrete characters was compiled for all taxa (Table S1, S2).

### Alignment and phylogenetic analyses

The three genes used were not length variable and the unambiguous alignment was based on conservation of amino acid reading frame. The concatenated alignment consisted of 1647 base pairs. The genes and the morphological data matrix were combined and analysed simultaneously in a Bayesian framework using MrBayes ver. 3.0b4 [39] with partition specific model settings; a separate HKY85+Γ+I model was assigned to each of four partitions of the molecular data defined as; 12posCO1, 3posCO1, 12posH3/Wingless, 3posH3/Wingless. The partitions and assigned model is by necessity a balance between reality and avoiding assigning more parameters than limited data can possibly estimate. For the morphological matrix a Markov k model+Γ [40] was used, accounting for the fact that only parsimony informative characters were scored. It was recently shown that model-based phylogenetic inference forcing differently evolved data to a uniform model is hazardous [41]. In particular, estimating the branch length as an average from the total data may yield erroneous likelihood calculations for each partition. Consequently branch lengths were unlinked and independently estimated for all five partitions, avoiding the potential pitfall. Four independent Markov Chain Monte Carlo runs with 10 million generations each were sampled every 1000 generation. The first 2 million generations were discarded in each run as burn-in and the last 8000 sampled trees were pooled from the four runs and summarized, identifying the topology with highest posterior probability. Model parameter estimations are given in Table S4. Proper mixing of chains, acceptance probabilities and convergence of likelihood values, tree topologies, branch lengths and model substitution parameters were checked in the four runs before the samples were pooled. Clade support values and posterior probability of topologies were calculated as the frequency of each clade/topology among the sampled trees. We also ran a parsimony analysis on the total evidence data with all characters equally weighted and unordered. Implicit enumeration in TNT [42] ensured finding the shortest tree with 15 taxa in reasonable time. Of the 1693 characters, 290 were parsimony informative (CO1: 152, H3: 39, Wingless: 53, Morphology: 46).

### Character reconstruction

Suction cup diameter S1–S4 (see fig. 4) was measured for five individuals of each species where fresh material was available. For S4 an average of 10 cups was calculated. Measurements and counting of S4 cups were carried out by digital photo (Kodak Megaplus camera Model 1.6i) through an Olympus microscope at 64× and imported to the image analysis software Optimas 6.5 (Media Cybernetics, 1987–1999). To accommodate uncertainty in the phylogeny reconstruction, character optimizations were done on all tree topologies that together cumulated a posterior probability of >95%, ordered in decreasing posterior probability from the Bayesian analysis. Discrete morphological characters were mapped using Fitch optimization, while continuous characters were optimized as additive using Farris optimization as implemented in TNT [42].

## Supporting Information

Supporting Text S1 Supporting text deriving from first principles the effect of a modified surface to the attachment time and suction force of mechanically working suction cups.
(0.05 MB DOC)

Figure S1 Phylogram from Bayesian analysis with branch-lengths estimated from the most variable partition, 3:rd codon positions of CO1. Scale-bar = expected number of substitutions per site. Note the recent divergence of the kishii/japonicus species pair.
(3.74 MB TIF)

Table S1 Description of morphological characters used in the phylogenetic analyses.
(0.04 MB DOC)

Table S2 Morphological character matrix of the 46 characters used in the phylogenetic analyses (see Supplementary Table S1).
(0.03 MB DOC)

Table S3 Collecting locality of sequenced specimens and accession numbers to sequences in GeneBank. CO1 (Cythochrome Oxidase I, 805bp), H3 (Histone 3, 376bp), Wnt (Wingless, 466bp). AF-numbers are from Miller (2003; reference 26 of main article).
(0.03 MB DOC)

Table S4 Model parameter estimations from the 4x8000 last samples of the Markov Chain in the Bayesian analysis. TL = tree length, kappa =  transition/transversion parameter, pi(A) = stationary frequency of adenine, alpha =  shape parameter of the gamma distribution, pinvar = proportion of invariable sites. Data partitions {1} = morphology, {2} = 12pos CO1, {3} = 3pos CO1, {4} = 12posH3&Wingless, {5} = 3posH3&Wingless. Morphology was given a Markov k model +Γ accounted for scoring only parsimony informative characters and each of the four molecular partitions were given a separate HKY85+ Γ+I model. Branchlengths were estimated separately for each of the five partitions.
(0.03 MB DOC)

Table S5 Measurements of male suction cups and size and density of female elytral structures in Acilius kishii (non-sulcate females) and A. japonicus (sulcate females). N = number of measured individuals over which the average is presented. S1–S4 suction cups according to Fig. 4. Sdi = distance over sulci, Sde = distance between sulci, (i.e. density), MPdi = diameter of macropunctures, MPde = distance between macropunctures (i.e. density). All values in mm. For S4, MPdi and MPde 10, 20 resp. 20 for each individual was measured. Sdi is presented as the average of the smallest to the widest sulcus.
(0.02 MB DOC)

Video S1 The movie shows three mating attempts including the two species Acilius sulcatus and Acilius canaliculatus, where the first two attempts are followed by matings, while in the third the female escapes.
(7.61 MB MOV)
