# Supplementary material for: Phylogeny of Diving Beetles Reveals a Coevolutionary Arms Race between the Sexes
Source: PLoS One. 2007 Jun 13;2(6):e522. doi: 10.1371/journal.pone.0000522 (PMC1885976; doi:10.1371/journal.pone.0000522)
Supplement: Supporting Text S1 — Supporting text deriving from first principles the effect of a modified surface to the attachment time and suction force of mechanically working suction cups. (0.05 MB DOC) [file pone.0000522.s001.doc]

**Supporting text**

It has long been recognised through simple experiments that the male suction cups in diving beetles attach firmly a smooth surface like the glass of an aquarium or a mounting slide under a microscope, but very poorly on anything less smooth like surfaces with depressions or pilosity [R1-R5]. Below we provide a derivation from first principles (physical laws of Newton and Poiseuille) explaining these observations, and we develop further upon the effect of larger or smaller suction cups on uneven surfaces.

The suction force *F* of a suction cup is [R6]

(1) $F=(p_{1}-p_{2})A$,

where *p*_1_ and *p*_2_ are the pressures outside and inside the cup respectively and

*A* is the area of the cup. The attachment time *t* of the cup is

(2) $t=\frac{\Delta V}{f}$,

where $\Delta V=V_{\max}-V_{\min}$ is the volume difference of the cup between the

time of attachment and the time it loses its grip, and *f* is the volume flow into

the cup. The flow can be written as

(3) $f=\frac{(p_{1}-p_{2})}{R}$,

where *R* is the flow resistance. This resistance depends linearly on the viscosity *η* of the medium (for water at 20°C this is 1.003 mPa s) and it depends strongly on the size of the channel where it is flowing. In the case of a circular channel (a tube) of radius *r* and length *L* the resistance is [R7]

(4) $R=\frac{8\eta L}{\pi r^{4}}$.

Note the very strong dependence on the radius, *r^4^*. The suction cup attaching to a surface can be modelled as a number of channels (*N*) around the circumference of the cup. The channel length *L* is the width of the suction ring and *r* is the radius

of the largest channels where water leaks into the cup. Because of the strong

dependence on the radius only a few channels must be taken into account. By combining the equations the attachment time can be considered

(5) $t=\frac{8A\Delta V\eta L}{\pi NFr^{4}}$.

The equation can be modified to

(6) $F=\frac{8A\Delta V\eta L}{\pi Ntr^{4}}$.

This means that the most important factor for the attachment time and suction force is the radius of the leakage channels. If the radius is doubled in size the attachment time and suction force is decreased by sixteen times! This is the effective difference between females with smooth elytra and those with punctured or setose furrowed elytra. Table S5 relates the scale of suction cups and structures on the female elytra and show that the small cups of males and the diameter and density of macropunctures on the elytra are closely scaled whereas the width of and distance between the female elytral sulci are of the same order of magnitude as the largest male suction cups. Since these structures occur on the surface of attachment for the suction cups, the channel effect modelled above is a necessary consequence (this need not be so if the structures were to occur on a scale orders of magnitude different from that of the suction cups).

Next the effect of suction cups of different size can be considered. A larger suction cup has a larger volume and area, which increases *t*. If it is assumed that the suction cup describes a hemisphere at *V_max_* and is elastic enough to be pressed almost completely flat (V_min_ ≈ 0), then the following associations apply

(7) $A=\pi a^{2}$, $\Delta V=\frac{2\pi a^{3}}{3}$,

where *a* is the radius of the suction cup. Eq. 5 then becomes

(8) $t=\frac{16\pi}{3}\frac{\eta La^{5}}{NFr^{4}}$.

*a^5^* indicates that increasing the suction cup radius will increase attachment time even more than reducing *r*. However, we have to account for that with larger suction cups, fewer will fit a given surface *S* (the male palette formed by the three enlarged protarsal segments). If a hexagonal pattern is assumed (the densest way to arrange circles on a surface) which is reasonable for most suction cup arrangements on diving-beetle feet, the number of cups is

(9) $M=\frac{S}{2a^{2}\sqrt{3}}$.

To incorporate the entire male palette, *S*, in Eq. 8, *F*

must to replaced by *F/M* since the total force *F* is shared between the cups, The attachment time is then

(10) $t=\frac{8\pi}{3\sqrt{3}}\frac{\eta LSa^{3}}{NFr^{4}}$.

This reduces the impact of *a* to *a*^3^. Nevertheless, this means that on a smooth surface, larger suction cups are more effective (attachment time and suction force), because *r* is constant (unless the suction cups shrink to the microscopic level of unevenness of a smooth surface). This conclusion is confirmed with experimental evidence (R8), where the largest 2 cups were capable of lifting a larger weight than the approx. 300 small cups, measured on the foot of *Dytiscus alaskanus* and attached to a smooth disc. This also explains the ancestral pattern of the outgroup with few large cups when the female elytron is smooth, and is an indirect physical evidence that the female change predated the change in the male since selection for better grip could not have been towards smaller cups if the female was smooth. On a surface that is uneven on a larger scale, small suction cups can reduce *r* by attaching on top of, in, or between the structures (e.g. the smallest cups on a macropunctured surface, see table S5), and because of the strong dependence on *r*, this can be advantageous despite simultaneous reduction of the suction cup radius. If it is advantageous depends on a balance between how much to the radius must be reduced compared to how much this reduces *r*. This cannot be easily predicted without examining details of the structure. However, it is reasonable to assume that for a given structure, a large suction cup could be selected for to become larger because it needs to be reduced to a very much smaller size before *r* starts to decrease, while increasing in size always increase *t* with the power of 3. At the same time, for the same structure, a smaller suction cup can be selected for to become smaller because it is in the range where *r* directly reduces with a small decrease in size. This can explain the bi-directional transformation of male suction cups with the largest three becoming larger and the smaller decreasing in size but increasing in number. The effect of differentiated suction cups on a palette cannot be described with a simple formula like the ones above. But we can, on the specific case of a smooth surface, or a rugged surface where the structures are much smaller than the suction cups, approximate the number of leakage channels with,

(10) $N=\frac{2\pi a}{d}$

where *d* is the average distance between the major leakage channels. This accounts for that a longer circumference means more leakage channels. We can then write

(11) $t=\frac{4}{3\sqrt{3}}\frac{\eta LSda^{2}}{Fr^{4}}$,

which is a good approximation of the largest suction cups (*a* = 0.5mm) on elytra with only macropunctation (*d*=0.03-0.06mm, *r* ≈0.01-0.03mm). The influence of *a* has here been reduced to the power of 2 when the effect on *N* has been considered.

**Supporting references**

R1. Plateau F (1872) Un mot sur le mode d'adhérencé des mâles de Dytiscides aux femelles pendant l'acte de l'accouplement. Ann Soc Ent Belg 15: 205-212.

R2. Euscher H (1910) Das chitinskelett von Dytiscus marginalis. Dissertation, Marburg, Der Universität Marburg.

R3. Guignot F (1931-33) Les Hydrocanthares de France. Toulouse, Les Frères Douladoure. 1057p.

R4. Frankenberg G von (1935) Der Haftapparat der Dytiscus und Acilius männchen *Mikrokosmos* 29: 143-148.

R5. Naumann H (1955) Der Gelbrandkäfer. A. Ziemsen Verlag, Wittenberg Lutherstadt. 80p.

R6. Newton I (1687) Philosophiæ Naturalis Principia Mathematica. Joseph Streater for the Royal Society, London.

R7. Poiseuille JLM (1840-41) Recherches experimentales sur le mouvement des liquides dans les tubes de très-petite diamètres. CR Acad Sci Paris 11: 961-967, 1041-1049, 12: 112-115.

R8. Aiken RB, Khan A (1992) The adhesive strength of the palettes of males of a boreal water beetle, Dytiscus alaskanus Browne, J.Balfour (Coleoptera, Dytiscidae). Can J Zool 70: 1321-1324.
