# Supplementary figures and images for: Phylogeny of Diving Beetles Reveals a Coevolutionary Arms Race between the Sexes
Source: PLoS One. 2007 Jun 13;2(6):e522. doi: 10.1371/journal.pone.0000522 (PMC1885976; doi:10.1371/journal.pone.0000522)

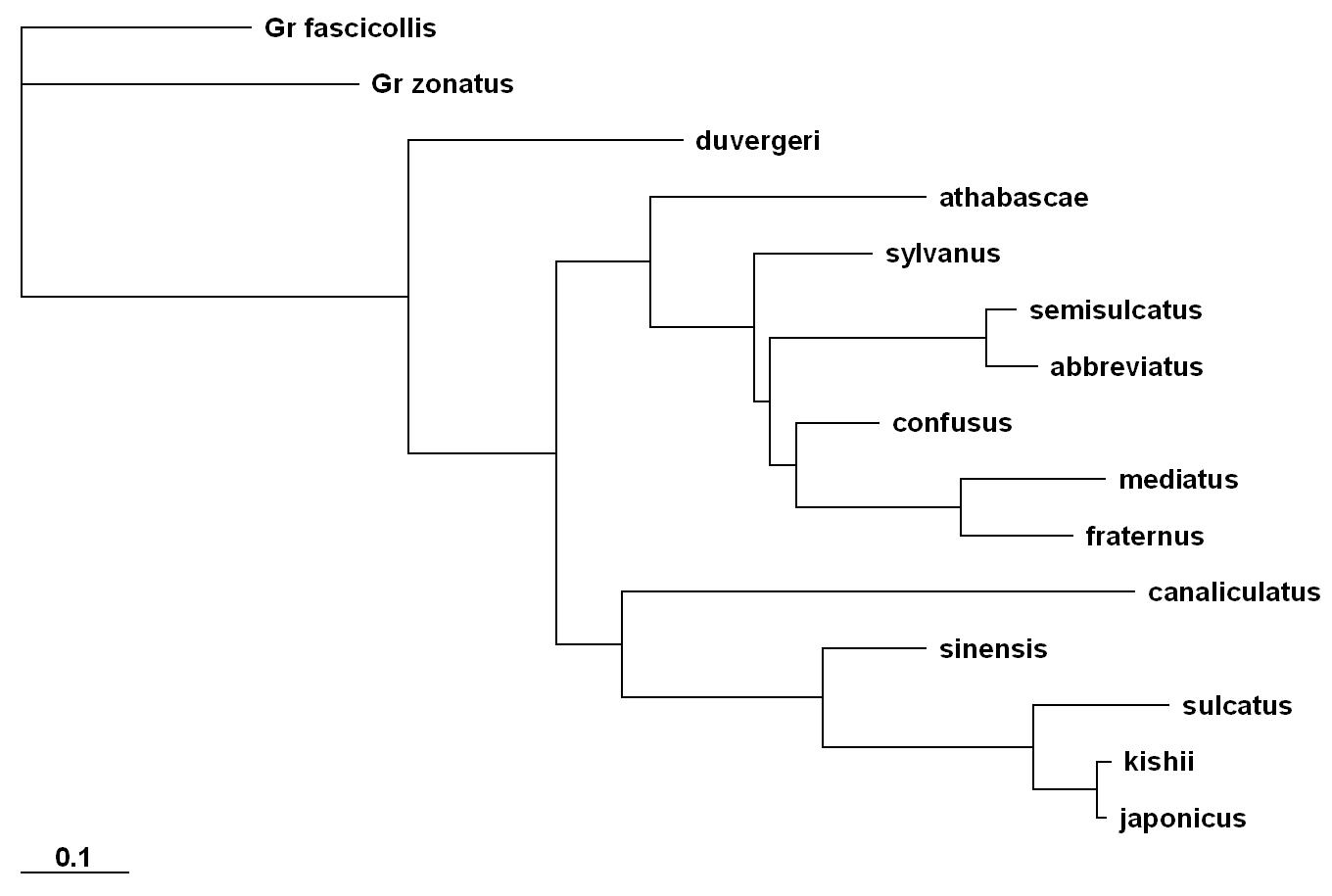

Supplement: Figure S1 — Phylogram from Bayesian analysis with branch-lengths estimated from the most variable partition, 3:rd codon positions of CO1. Scale-bar = expected number of substitutions per site. Note the recent divergence of the kishii/japonicus species pair. (3.74 MB TIF) [file pone.0000522.s002.tif]
