# Supplementary material for: Phylogeny of Diving Beetles Reveals a Coevolutionary Arms Race between the Sexes
Source: PLoS One. 2007 Jun 13;2(6):e522. doi: 10.1371/journal.pone.0000522 (PMC1885976; doi:10.1371/journal.pone.0000522)
Supplement: Table S1 — Description of morphological characters used in the phylogenetic analyses. (0.04 MB DOC) [file pone.0000522.s003.doc]

Female genitalia

1 Shape of subapical portion of gonocoxa

0 parallell and close together all the way

1 greatly enlarged before split of apical portion forming a "hip"

2 slightly enlarged during a longer section

2 Apical separated gonocoxal lobes

0 parallell and close together, inner face flat

1 diverging and distinctly set apart, inner face not flat

3 Rami extension

0 rami extending beyond the split between gonocoxal lobes

1 rami not extending beyond the split between gonocoxal lobes

4 Shape of apical end of laterotergites

0 flat and scleroterised

1 irregular shape, soft tissue on upper surface

5 Gonocoxal lobes

0 Not prolonged nor enlarged apically, apical portion not longer than wide

1 prolonged and enlarged apically, apical portion at least twice as long as wide

Male genitalia

*Median lobe*

6 Median lobe apex, ventral view: deflexed edges around lateral margins

0 absent

1 present

7 Median lobe apex, ventral view: parallel ridges along medial axis

0 joining before apex

1 not joining before apex

8 Median lobe apex, ventral view: single median axis before apex

0 yes

1 no

9 Median lobe outer half, ventral view

0 flat, sides not flexed downward

1 sides flexed downwards giving lobe a triangular shape

10 Median lobe apex: lateral edges

0 lateral edges flexed backwards-inwards, in a rounded fashion

1 lateral edges flat

2 lateral edges flexed upwards

11 Median lobe apex

0 apex pointed*

1 apex blunt

2 bifid

* The trifid apex of *Graphoderus* is not interpreted as being homologous as the outer two points are made up of non-sclerotized tissue and the aedeagus is thus interpreted as simple

12 Median lobe lateral view

0 more or less straight to the apex

1 ascending towards the apex

2 ascending well before apex, there after gently curved back

13 Median lobe apex dorsal side: median ridge

0 simple

1 forming a channel towards apex

14 Median lobe base dorsal side: split opening

0 narrow

1 widening inwards

15 Median lobe basally, ventral view: lateral muscle attachment flangs

0 more or less vertical basally

1 flangs basally with a 45d - horizontal orientation

*Parameres*

16 Colour of parameres basicointernally

0 concolourous with surroundings

1 restricted area piceous to infuscated

17 Distinct longitudinal, extra sclerotized, band basicolaterally on parameres

0 present

1 absent

Male tarsi

18 Male protarsal claws

0 anterior and posterior claws subequal

1 anterior claw distincly longer and thicker than posterior

19 Male anterior protarsal claw length

0 clawlength/total bodylength less than 0.04

1 claw very long and falsiform clawlength/total bodylength more than 0.05

20 Male anterior mesotarsal claw

0 ventral side not expanded

1 ventral side expanded

21 Male protarsal adhesive discs

0 Size of protarsal discs subequal (S1/S4<3.5)

1 Size of protarsal discs very unequal; three big and many small (S1/S4>10)

22 The three large male protarsal discs

0 subequal (S1/S3<1.65)

1 the basal one much larger than the other two (S1/S3>2.0)

23 Small adhesive discs on anterior corner of 1:st protarsal segment

0 present

1 absent

24 Distribution of the small adhesive discs on protarsus

0 distributed evenly on 2:nd and 3:rd segment and laterodistally on 1:st segment

1 forming two unequal groups of discs widely set apart by an empty median field

25 Sizevariation of small adhesive discs on protarsus

0 all small discs subequal in size

1 diameter of small discs increase towards outer margin

26 Hairfringe around protarsal palette

0 simple straight hairs

1 hair recurved apically

27 Male mesotarsal segments 1 and 2 ventrally

0 adhesive discs present on external side

1 adhesive discs absent

28 Male mesotarsal segment 3 ventrally

0 adhesive discs present on external side

1 adhesive discs absent

29 Male mesotarsal segment 1 ventrally

0 adhesive discs present on whole segment

1 adhesive discs absent at least on internal half

30 Male mesotarsal segments 1-3 ventrally

0 tufts of hair absent

1 tufts of hair present on internal half

Punctuation

31 Rough and dense dorsal punctuation on elytra

0 absent

1 present

32 Rough and dense punctuation ventrally on metacoxal plate, metasternal wing, and abdominal sternites

0 absent

1 present

33 Dorsal punctuation on elytra

0 rounded

1 crescentic

Elytra

34 Width of epiplura at level of 2:nd abdominal sternite

0 1/3:rd or less than width of 2:nd abdominal sternite

1 about half the width of 2:nd abdominal sternite

Female elytra & pronotum

35 Female elytra

0 smooth, non-sulcate

1 sulcate, 4 furrows set with hair on each elytra

36 Anterior length of innermost sulcus

0 not reaching as far anteriorly as the other sulci

1 the four sulci equal in length anteriorly

37 Colour of hair covering the sulci

0 light

1 dark

38 Surface of pronotum sexually dimorphic

0 female not very different from male

1 female with obvious depression laterally often set with hair*

* In *sulcatus* always abundant, in *japonicus* always a few hairs, in *kishii*, *canaliculatus* and *sinensis* some individuals with few hairs, some without hair

Colouration

39 Distinct light subapical fasciae on elytra

0 absent

1 present

40 Coloration of head

0 M-mark present

1 M-mark present without "legs"

2 head rufous, M-mark indistinct or absent

41 Coloration in front of M-mark on head

0 black mark forming an angle anterior to M-mark

1 no black mark anterior to M-mark (sometimes two black dots)

42 ventral colouring; metafemur

0 entirely testaceous

1 infuscated at base

2 entirely infuscated, except apically

43 ventral colouring; 1:st abdominal segment

0 testaceous

1 infuscated

44 ventral colouring; 2:nd abdominal sternite

0 at least with a lateral yellow spot

1 entirely infuscated

45 ventral colouring; metacoxa and metasternum

0 testaceous

1 rufous to infuscated

Bodyshape

46 Bodywidth/bodylength, lateral edges of elytra

0 body not excessively broad <0.59, lateral edges of elytra narrow

1 body markedly very broad >0.60, lateral edges of elytra widened
