# Supplementary material for: Phylogeny of Diving Beetles Reveals a Coevolutionary Arms Race between the Sexes
Source: PLoS One. 2007 Jun 13;2(6):e522. doi: 10.1371/journal.pone.0000522 (PMC1885976; doi:10.1371/journal.pone.0000522)
Supplement: Table S2 — Morphological character matrix of the 46 characters used in the phylogenetic analyses (see Supplementary Table S1). (0.03 MB DOC) [file pone.0000522.s004.doc]

1 2 3 4

1 1 1 1 1

G. fascicollis 20100 0-011 01001 01000 00000 00000 00-00 --000 00000 0

G. zonatus 20100 0-011 00001 01000 00000 00000 00-00 --000 00000 0

duvergeri 21100 01101 00011 11000 10110 00100 11100 --001 10001 0

canaliculatus 11111 01102 10001 11111 11110 10011 11101 10100 1000- 1

sinensis 10111 11100 20111 11111 11111 11111 11101 10102 10001 1

sulcatus 11111 11100 20011 11111 11111 10011 11101 10100 01101 1

kishii 11111 11100 20111 11111 11111 10011 11110 --100 01101 1

japonicus 11111 11100 20111 11111 11111 10011 11111 10100 01101 1

athabascae 00001 00111 11000 01110 11110 11111 11101 10000 10000 0

sylvanus 00001 00011 11000 01000 11110 11111 11101 00000 10000 0

confusus 00001 00110 12011 01000 11110 11111 11101 01012 12111 0

mediatus 21101 00110 11011 00000 10110 01110 11000 --010 12111 0

fraternus 00001 00010 12011 00000 10110 01110 11000 --012 12111 0

semisulcatus 00001 00110 11000 01101 11110 11111 11101 01010 11101 0

abbreviatus 00001 00110 11000 01100 11110 11111 11101 01010 1--0- 0
