# Supplementary material for: Phylogeny of Diving Beetles Reveals a Coevolutionary Arms Race between the Sexes
Source: PLoS One. 2007 Jun 13;2(6):e522. doi: 10.1371/journal.pone.0000522 (PMC1885976; doi:10.1371/journal.pone.0000522)
Supplement: Table S3 — Collecting locality of sequenced specimens and accession numbers to sequences in GeneBank. CO1 (Cythochrome Oxidase I, 805bp), H3 (Histone 3, 376bp), Wnt (Wingless, 466bp). AF-numbers are from Miller (2003; reference 26 of main article). (0.03 MB DOC) [file pone.0000522.s005.doc]

**Species Locality CO1 H3 Wnt**

Graphoderus fascicollis New York DQ275310 DQ275318 AF392015

Graphoderus zonatus Sweden DQ275311 DQ275319 AF392017

Acilius duvergeri Sardinia DQ275300 DQ275316 DQ275328

Acilius canaliculatus Sweden DQ275298 DQ275314 DQ275330

Acilius sulcatus Sweden DQ275308 DQ275324 AF392000

Acilius kishii Japan DQ275305 DQ275321 DQ275327

Acilius japonicus Japan DQ275302 DQ275320 DQ275326

Acilius japonicus Japan DQ275303 - -

Acilius japonicus Japan DQ275304 - -

Acilius athabascae Alberta DQ275297 DQ275313 DQ275332

Acilius sylvanus New York DQ275309 DQ275325 AF392001

Acilius confusus Maryland DQ275299 DQ275315 DQ275331

Acilius mediatus New York DQ275306 DQ275322 AF391998

Acilius fraternus New York DQ275301 DQ275317 DQ275329

Acilius semisulcatus New York DQ275307 DQ275323 AF391999

Acilius abbreviatus California DQ275296 DQ275312 DQ275333
