# Supplementary material for: Phylogeny of Diving Beetles Reveals a Coevolutionary Arms Race between the Sexes
Source: PLoS One. 2007 Jun 13;2(6):e522. doi: 10.1371/journal.pone.0000522 (PMC1885976; doi:10.1371/journal.pone.0000522)
Supplement: Table S4 — Model parameter estimations from the 4x8000 last samples of the Markov Chain in the Bayesian analysis. TL = tree length, kappa = transition/transversion parameter, pi(A) = stationary frequency of adenine, alpha = shape parameter of the gamma distribution, pinvar = proportion of invariable sites. Data partitions {1} = morphology, {2} = 12pos CO1, {3} = 3pos CO1, {4} = 12posH3&Wingless, {5} = 3posH3&Wingless. Morphology was given a Markov k model +Γ accounted for scoring only parsimony informative characters and each of the four molecular partitions were given a separate HKY85+ Γ+I model. Branchlengths were estimated separately for each of the five partitions. (0.03 MB DOC) [file pone.0000522.s006.doc]

95% Cred. Interval

Parameter Mean Variance Lower Upper Median

TL{1} 2.212734 0.095013 1.659000 2.862000 2.197000

TL{2} 1.895999 0.199090 1.132000 2.870000 1.857000

TL{3} 3.889841 0.186325 3.121000 4.801000 3.862000

TL{4} 2.508071 0.262258 1.584000 3.598000 2.477000

TL{5} 1.009366 0.016018 0.811000 1.311000 0.992000

kappa{2} 80.24577 2171.532 30.00548 206.4486 73.81058

kappa{3} 18.23334 8.764947 13.12749 24.56971 18.00226

kappa{4} 5.047674 11.79944 1.372264 13.44133 4.251979

kappa{5} 3.737930 0.348663 2.709818 5.025459 3.692989

pi(A){2} 0.242388 0.000333 0.207355 0.279017 0.242162

pi(C){2} 0.193091 0.000248 0.163503 0.225213 0.192682

pi(G){2} 0.187679 0.000272 0.156973 0.221408 0.187140

pi(T){2} 0.376842 0.000406 0.337691 0.416748 0.376634

pi(A){3} 0.415894 0.000501 0.372035 0.459915 0.415799

pi(C){3} 0.075236 0.000052 0.061959 0.090143 0.074911

pi(G){3} 0.037166 0.000023 0.028392 0.047027 0.036932

pi(T){3} 0.471704 0.000464 0.429584 0.514385 0.471482

pi(A){4} 0.303522 0.000368 0.266678 0.341640 0.303266

pi(C){4} 0.234690 0.000311 0.201035 0.270197 0.234386

pi(G){4} 0.266792 0.000338 0.231278 0.303577 0.266475

pi(T){4} 0.194996 0.000281 0.163143 0.228705 0.194720

pi(A){5} 0.200984 0.000351 0.164982 0.238517 0.200648

pi(C){5} 0.318282 0.000467 0.276597 0.360969 0.318010

pi(G){5} 0.241850 0.000389 0.204239 0.281663 0.241502

pi(T){5} 0.238884 0.000374 0.202259 0.277733 0.238385

alpha{1} 23.99448 188.2803 2.421848 48.35852 23.47756

alpha{2} 0.240245 0.010915 0.114629 0.527774 0.212096

alpha{3} 1.382926 0.270581 0.764055 2.667816 1.266136

alpha{4} 0.083091 0.001086 0.059882 0.185586 0.073460

alpha{5} 20.71916 207.5944 1.375077 48.08177 18.75155

pinvar{2} 0.888064 0.000655 0.831361 0.929665 0.890731

pinvar{3} 0.075797 0.002625 0.003329 0.187313 0.068293

pinvar{4} 0.927531 0.000425 0.881659 0.961957 0.929435

pinvar{5} 0.299074 0.005854 0.102000 0.417499 0.310149
