# Supplementary material for: Phylogeny of Diving Beetles Reveals a Coevolutionary Arms Race between the Sexes
Source: PLoS One. 2007 Jun 13;2(6):e522. doi: 10.1371/journal.pone.0000522 (PMC1885976; doi:10.1371/journal.pone.0000522)
Supplement: Table S5 — Measurements of male suction cups and size and density of female elytral structures in Acilius kishii (non-sulcate females) and A. japonicus (sulcate females). N = number of measured individuals over which the average is presented. S1–S4 suction cups according to Fig. 4. Sdi = distance over sulci, Sde = distance between sulci, (i.e. density), MPdi = diameter of macropunctures, MPde = distance between macropunctures (i.e. density). All values in mm. For S4, MPdi and MPde 10, 20 resp. 20 for each individual was measured. Sdi is presented as the average of the smallest to the widest sulcus. (0.02 MB DOC) [file pone.0000522.s007.doc]

S_di_ S_de_ S1 S2 S3 MP_di_ MP_de_ S4

N 5 5 5 5 5 5 5 5

A. kishii - - 1.101 0.413 0.335 0.048 0.041 0.029

A. japonicus 0.699 0.395-0.960 1.111 0.460 0.387 - - 0.032
